# PRC2: an epigenetic multiprotein complex with a key role in the development of rhabdomyosarcoma carcinogenesis

**DOI:** 10.1186/s13148-021-01147-w

**Published:** 2021-08-09

**Authors:** Stefano Zoroddu, Irene Marchesi, Luigi Bagella

**Affiliations:** 1 Department of Biomedical Sciences, University of Sassari, Viale San Pietro 43/b, 07100 Sassari, Italy; 2 Kitos Biotech Srls, Tramariglio, Alghero, SS Italy; 3 Sbarro Institute for Cancer Research and Molecular Medicine, Center for Biotechnology, College of Science and Technology, Temple University, Philadelphia, PA USA

**Keywords:** Histone modification, Epigenetics, Rhabdomyosarcoma, Cancer, Methyltransferase, EZH2

## Abstract

Skeletal muscle formation represents a complex of highly organized and specialized systems that are still not fully understood. Epigenetic systems underline embryonic development, maintenance of stemness, and progression of differentiation. Polycomb group proteins play the role of gene silencing of stemness markers that regulate muscle differentiation. Enhancer of Zeste EZH2 is the catalytic subunit of the complex that is able to trimethylate lysine 27 of histone H3 and induce silencing of the involved genes. In embryonal Rhabdomyosarcoma and several other tumors, EZH2 is often deregulated and, in some cases, is associated with tumor malignancy. This review explores the molecular processes underlying the failure of muscle differentiation with a focus on the PRC2 complex. These considerations could open new studies aimed at the development of new cutting-edge therapeutic strategies in the onset of Rhabdomyosarcoma.

## Introduction

**Fig. 1:**
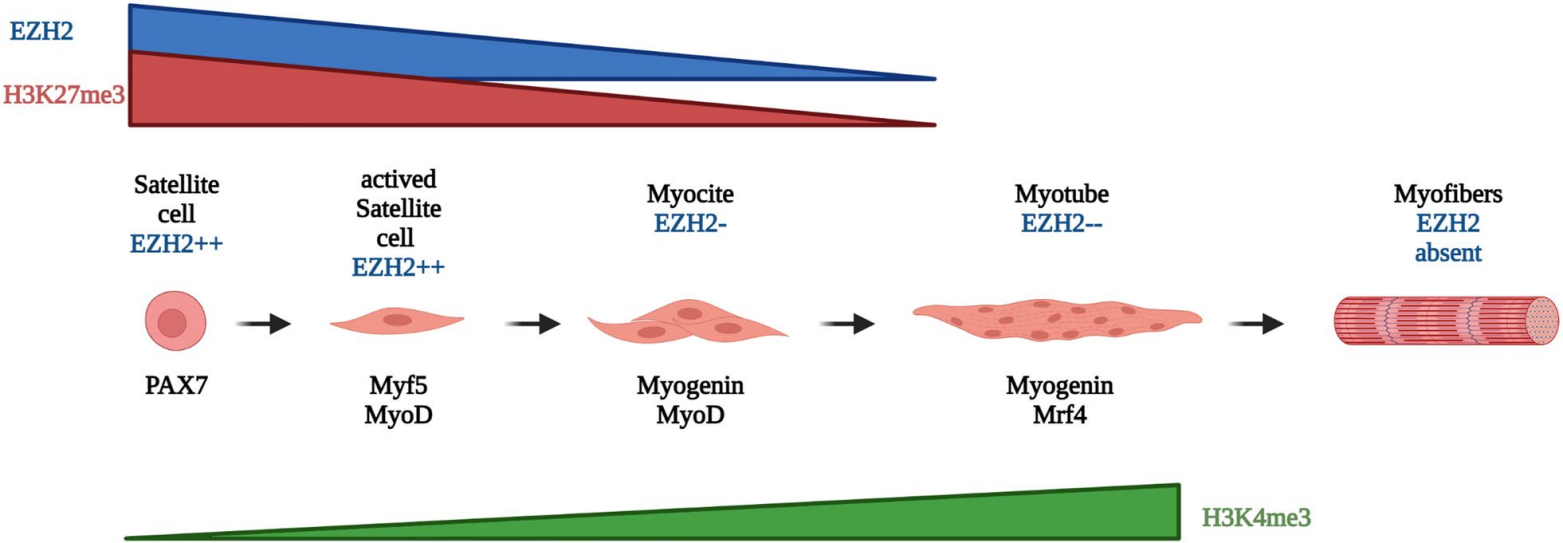
Myoblastic differentiation. During the early stages, satellite cells are activated, they proliferate and express MyoD, initiating transcription of muscle-specific genes required for early differentiation. As myogenesis proceeds, some activated satellite cells return to quiescence and renew the satellite cell reserve population, whereas others exit the cell cycle to undergo further differentiation. Those post-mitotic myocytes show a shift in gene expression that allows their fusion to form multinucleated myotubes capable of undergoing terminal differentiation. During these phases, EZH2 expression decreases dramatically coupled with a decrease in lysine 27 methylation of histone H3. Conversely, methylation of lysine 4 of histone H3 increases, allowing RNA polymerase access to muscle-specific genes

Myogenesis is a complex multi-stage process that requires highly precise, controlled regulation, which occurs both during embryonic development and during muscle regeneration and repair. The process begins with the mesodermal progenitors and culminates with differentiation and maturation into myofibres, which build muscle and muscle innervation through the newly formed neuromuscular junction [1]. The differentiation process is hierarchically controlled under the precise control of a main regulator present in specific phases of temporal and spatial development [2]. Myogenic regulatory factors (MRFs) are a family of transcription factors whose function and activity represent a series of molecular switches that determine muscle differentiation. They are represented by a group of four specific muscle proteins, including MyoD, Myf5, Myogenin and Myogenic Regulatory Factor 4 (MRF4). MRFs operate by regulating proliferation, activating muscle-specific sarcomeric genes preceded by an irreversible arrest of the cell cycle of precursor cells [2]. Each of the MRFs can act as a major regulator of myogenesis, however, their expression levels are finely modulated to ensure proper muscle maturation progression. MRFs contain a basic helical domain (bHLH) that gives the ability to recognize the E-box sequence, which is found in both the promoter and the muscle-specific gene enhancer sequences, inducing their transcriptional activation and myogenesis progression [3]. The first factor that has been identified is MyoD, it has a crucial role in initiating the myogenic differentiation program by modulating the activity of over 300 muscle-specific genes, such as myogenin, M-cadherin, myosin heavy (MHC), light chains (MLC), and muscle creatine kinase (MCK). Binding of MyoD to DNA is achieved by heterodimerization with other non-myogenic bHLH proteins, such as E2A gene products (E12, E47) [4]. In target gene promoters, MyoD heterodimers recruit a multiprotein complex consisting of SWI/SNF, pTEFIIb, and the p300 histone acetyltransferases, PCAF. This complex induces histone acetylation and changes in nucleosomal conformation. In addition, it is involved in promoting transcription elongation through phosphorylation of the carboxy-terminal domain (CTD) of RNA polymerase II (RNA Pol II), converting the complex to the phosphorylated and active form, thereby promoting gene expression [5, 6]. Subsequently, another factor called Myf5 was identified, whose expression appears to be critical, together with MyoD, for the determination of the myogenic lineage then for myoblast formation, both of which can be considered as specification factors. MyoD appears to be involved in the terminal differentiation of myoblasts into myotubes, whereas Mrf4 shows a complex temporal expression suggesting a role in both determination and terminal differentiation of the myogenic lineage [7]. During embryogenesis, multiple extracellular signals, both inhibitory and stimulatory, induce pluripotent precursors of the paraxial mesoderm to become skeletal muscle cell precursors. These precursors, known as myoblasts, proliferate in response to MyoD and Myf5 (Fig. 1). Subsequently, they express the cyclin-dependent kinase inhibitor p21, exit the cell cycle, differentiate into myocytes, and begin to express late MRFs (myogenin and Mrf4) and muscle-specific genes such as myosin heavy chain (MYH) and creatine muscle kinase (MCK). Mononuclear myocytes in different body districts fuse together to form post-mitotic polynuclear myotubes and eventually organized into differentiated and highly specialized muscle fibers [8]. Factors that act as myogenic antagonists have been identified, binding directly to proteins and preventing interaction with MRF factors, or to MRFs such as MyoD, by blocking their ability to bind the E-box sequences of muscle-specific genes. Many of these inhibitors are themselves proteins in the bHLH family that includes Id, Twist, MyoR, and Mist-1. In contrast, other factors act as co-activators and co-repressors of myogenic transcription. Co-activating factors interact with transcription factors to activate muscle-specific gene expression; histone-modifying proteins, acetylases and methylases, SWI/SNF family chromatin remodeling factors, and TRAP/Mediator family proteins are among these factors. Co-repressor factors, such as histone deacetylases, negatively regulate muscle-specific gene expression by interacting with MyoD and Mef2 proteins [7]. The combined action of these transcription factors leads to muscle tissue formation and differentiation through the induction of precise molecular pathways.

## Rhabdomyosarcoma

Rhabdomyosarcoma (RMS) is the most common type of soft tissue sarcoma (STS). RMS is mainly observed in young patients, the most frequent subtypes are two: PAX-fusion negative or embryonic rhabdomyosarcoma (eRMS) and PAX-fusion positive or alveolar rhabdomyosarcoma (aRMS), which differ in both cytogenetic and molecular aspects [9]. eRMS is about 70% of childhood rhabdomyosarcoma and is most often found in the head and neck region and in the genitourinary tract [10]. aRMS is observed in about 30% of cases and manifests itself in the deep tissue of the extremities. eRMS particularly affects children, while aRMS occurs in both children and adolescents [11]. Embryonic RMS is not associated with a recurrent chromosomal rearrangement but, has a variety of genetic abnormalities, the most frequent being loss of heterozygosity on chromosome 11 at the 11p15.5 locus. Candidate genes at this genomic locus are p57 and IGF2, which are involved in muscle differentiation, arrest of cell proliferation, and enhancement of gene expression at the muscle level [9]. In addition, embryonic rhabdomyosarcoma cell lines and biopsy specimens carry activating mutations in the RAS oncogene. Activating mutations in N-RAS can occur in up to 20% of embryonal rhabdomyosarcoma cases, whereas mutations in H-RAS and K-RAS appear to be quite rare [12]. Alveolar RMS (aRMS) histologically appears similar to normal lung parenchyma [13]. It is characterized by the presence of one of two recurrent chromosomal translocations involving chromosomes 1 or 2 and chromosome 13: t (2;13) found in 55% of cases, or t (1;13) in 22% of cases. Because of these translocations, fusion of FKHR gene (FOXO1) on chromosome 13 with PAX 3 (chromosome 2) or PAX 7 (chromosome 1) occurs. In both cases this will result in the formation of oncogenic fusion proteins, in which the DNA-binding domain of the PAX gene is fused with the C-terminal transcriptional activation domain of the FOXO1 gene, resulting in increased transactivation functions compared to PAX3 and PAX7 alone [9]. Disruption of PAX genes leads to abnormal muscle development, suggesting a causal relationship between the translocation and the development of malignancy. In addition, the PAX3-FOXO1 translocation appears to have a poorer prognosis than PAX7-FOXO1 [14]. Recent studies have highlighted new subtypes of RMS: spindle cell sclerosing rhabdomyosarcoma emerged as a third subtype of pediatric RMS and a fourth pleiomorphic RMS subtype found exclusively in adults [15]. Current treatments on rhabdomyosarcoma use the PAX fusion state for risk assignment. The PAX fusion positive group denotes a high-risk subtype with a less favorable prognosis than those that are negative for PAX fusion [16, 17]. Treatment is multidisciplinary, includes surgery, chemotherapy and radiotherapy, these therapies have increased overall 5-year survival (OS) to about 70–90% for high-risk rhabdomyosarcoma and low-risk rhabdomyosarcoma, respectively. However, treatment toxicities significantly decrease quality of life [18, 19]. Overall, although the survival of rhabdomyosarcoma patients has improved considerably in recent years, a significant percentage still die from advanced disease [20]. The main causes of death are the presence of metastases, the site of origin, age of the patients, and the histological and genetic properties of the tumor. Considering these issues and the difficult efficacy of the oncological treatments used to date [9], numerous research efforts are currently focused on identifying new therapeutic target genes and drug combination strategies to combat rhabdomyosarcoma.

## Epigenetics and polycomb-group proteins

**Fig. 2:**
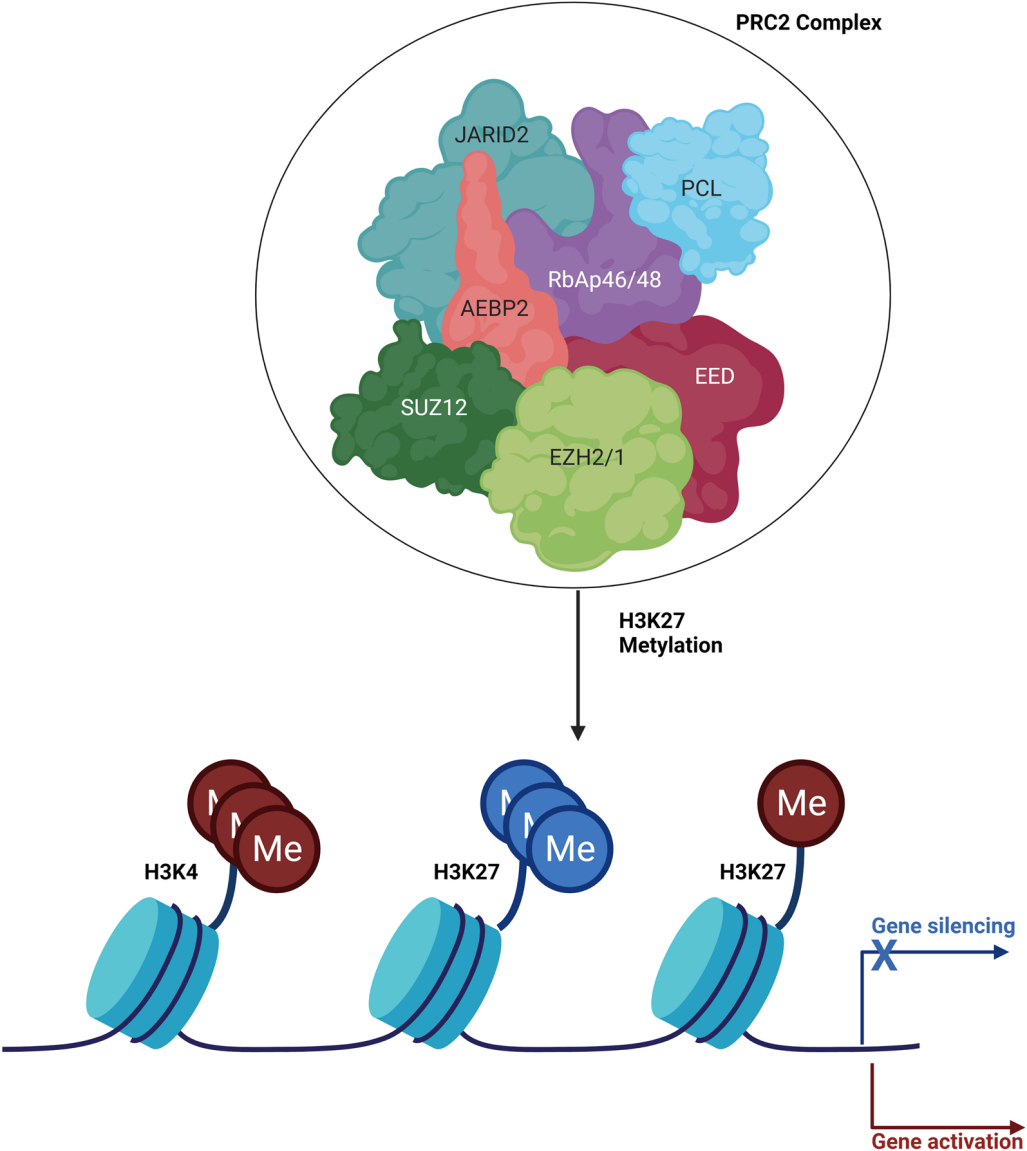
Polycomb Repressive Complex 2 (PRC2). PRC2 catalyzes the methylation of H3K27 through its enzymatic subunit EZH1 or EZH2. H3K27me3 correlates with gene silencing

A very important role in the onset and development of cancer is played by epigenetics which plays the crucial function of regulating the transcription of a very large number of genes. Epigenetics encompasses heritable structural and biochemical changes in chromatin without changing the DNA sequence [21]. Epigenetic mechanisms manipulate various physiological and pathological processes through the regulation of relevant gene expression by changing the accessibility of epigenetic codes to chromatin locally and globally [22]. Some of these genes are implicated in RMS so it is essential to focus on changes in chromatin condensation associated with the repression or over-expression of these genes especially during cell division. RMS cells are characterized by lacking of expression of differentiated myocyte genes, expressing markers of early myogenic differentiation and failing to stop proliferation [23]. The preservation of cellular identity requires intricate regulation of gene expression and strict control of transcriptional states over cell generations [24]. Polycomb group proteins (PcG) are important factors associated with chromatin modifications that contribute to the regulation of transcriptional repression [25]. PcG proteins have been identified in Drosophila melanogaster as responsible for the silencing of the homeopathic gene (Hox) and are also proteins present in humans as a demonstration of a conservation mechanism among eukaryotes [26–30]. The most studied multimeric PcG protein complexes are the Polycombic Repressive Complexes 1 and 2 (PRC1 and PRC2), they are essential for the precise and accurate regulation of development in many physiological systems, including skeletal muscle [31]. PRC1 is formed by BMI1, RING1A/B, CBX, and PHC subunits [32], while PRC2 is composed of EZH2, EED, SUZ12, and RbAp46 [23]. Both induce gene silencing either synergistically or by independently acting mechanisms [33, 34]. PRC2 catalyzes mono-, di- and trimethylation on lysine 27 on histone H3 and induces the recruitment of PRC1 (H3K27me1, H3K27me2 and H3K27me3) [24]. The link of PRC1 induces transcriptional repression of target gene through mono-ubiquitination of histone H2A lysine 119, by the RING1A or RING1B ubiquitin ligase catalytic subunit [35, 36]. Therefore, H3K27me3 can be considered the marker of PcG-mediated repression, whereas the PRC1 complex performs gene silencing [37]. PRC1 and PRC2 are also able to induce gene silencing independently of each other, but the synergistic mechanism is the most frequent. EZH1 is a homolog of EZH2, which gives rise to an alternative PRC2 complex (Fig. 2). However, data on this protein are sometimes conflicting [23]. The core of the complex forms distinct subcomplexes incorporating different combinations of partners, suggesting a role in the recruitment or regulation of PRC2 activity. Protein interaction data show segregation into two major subtypes of PRC2 named PRC2.1 [containing a PCL homolog (PCL1-3) along with EPOP (C17ORF96) or PALI (C10ORF12)] and PRC2.2 (containing JARID2 and AEBP2) [38]. The functions of the PRC2.1 and PRC2.2 subunits have been studied, however, it is still unclear whether they play a redundant role or whether they have mechanistically distinct roles in regulating PRC2 activity. However, some studies have highlighted important aspects of this issue, the first of which carried out on mouse embryonic stem cell (EMS) knockdown-PRC2.1-PRC2.2 lines showed that the two subunits are largely redundant in the pluripotent state [39, 40]. In contrast, a study in mice shows the need for both subunits for proper development [41]. These data suggest that the distinct functions of the two accessory subunits could be revealed by selectively depleting them during cell fate transitions. In recent years, genes coding for PRC2 subunits have been found mutated or deregulated in cancer. EZH2 and/or its closely related counterpart EZH1, are the catalytic subunits of the PRC2 complex. They guarantee a correct change of the transcriptome during development; therefore, mutations or alterations of their expression are related to the onset of cancer [23, 42–44]. Polycomb group proteins are epigenetic regulators of embryonic development and stem cell maintenance [45] and their deregulation contributes to cancer development [46]. Notably, it has been observed that in various cancers, including Rhabdomyosarcoma, there is over-expression of the catalytic subunit of the PRC2 complex, enhancer of zeste homolog 2 (EZH2), and its expression is linked to advanced disease stages and poor prognosis [47]. EZH2 activity is high in RMS and other tumor tissues, particularly in metastatic cancer. The PRC2 core sub-units, SUZ12 and EED, are associated with equimolar stoichiometry and are all necessary for the catalytic activity of the complex [45–54]. In this review we discuss the recent wave of interesting data that open new horizons in the complete understanding of the molecular mechanisms that regulate the function of PRC2 and to guide the development of new therapeutic strategies.

## Catalytic subunits of the PRC2 complex

Enhancer of zeste homolog 2 (EZH2) is the catalytic subunit of the PRC2 complex and is characterized by methyltransferase activity. It represses gene transcription by silencing target genes through methylation of lysine 27 of histone H3 (H3K27me3). This enzyme is responsible for silencing various genes involved in different processes such as cell cycle progression, apoptosis and differentiation and it is often deregulated in cancer [55].

The role of EZH2 during the cell differentiation processes is to prevent, in cell precursors, premature cell cycle arrest and activation of developmental genes, maintaining their proliferative potential. The dependence of differentiation processes on the proper cellular epigenetic landscape suggests that epigenetic abnormalities lead to altered gene expression and cellular physiology in several diseases [23]. The expression of EZH2 during development is finely regulated, it is specifically expressed in stem cells and undifferentiated cell progenitors, while its expression decreases during differentiation processes. A large number of human cancers, such as rhabdomyosarcoma, are associated with abnormally high levels of EZH2 compared to corresponding normal tissue. Furthermore, EZH2 expression is generally correlated with the development of a metastatic type of tumor and often behaves as a molecular biomarker of poor prognosis [52, 53]. It has been proposed that the role of EZH2 in cancer is related to its activity in promoting self-renewal and maintaining the undifferentiated state of cells. The target genes of EZH2 are generally oncosuppressor genes or those involved in cell differentiation; its deregulation promotes uncontrolled cell proliferation and thus cancer progression [52]. For example, abnormal up-regulation of EZH2 leads to transcriptional repression of the INK4b/ARF/INK4a locus and down-regulation of p16, p15, and p14, inhibitors of cyclin-dependent kinases (CDKs), resulting in uncontrolled proliferation and inhibition of apoptosis [48]. Finally, several differentiation factors are targets of PRC2, e.g., Gata, Sox, Fox, Pou, PAX components of Wnt, TGF-β, Notch, FGF, and retinoic acid pathways. EZH2-dependent silencing of these factors inhibits differentiation and promotes carcinogenesis [54, 56]. EZH2 has been shown to act as a negative regulator of skeletal muscle differentiation by promoting the proliferation of myogenic precursors. This function is the result of a direct, EZH2-dependent repression of genes related to myogenic differentiation [57]. The hypothesis that PRC2 has a repressive role on muscle-specific genes was confirmed with the establishment of an RD EZH2-knockdown cell line [58]. This cell line, in which EZH2 is downregulated, showed a partial recovery of the physiological muscle phenotype with the formation of some myotubes. Furthermore, gene and protein expression analysis showed that down-regulation of EZH2 leads to increased expression of MyoD and muscle-specific genes. Therefore, Ablation of EZH2 would induce restoration of MyoD binding and activation of muscle-specific genes. MyoD recruits p300, PCAF, SWI/SNF, and pTEFII to muscle-specific gene promoters to induce gene expression [59]. Several studies suggest that restoration of MyoD activity could lead to RNA Pol II phosphorylation, H3K27me3 demethylation and transcriptional activation resulting in partial recovery of the muscle phenotype [58]. These results demonstrate the crucial role of EZH2-dependent epigenetic alteration in rhabdomyosarcoma carcinogenesis and allow us to hypothesize that modulation of EZH2 may be a therapeutic target for the treatment of RMS.

EZH1 is still poorly studied but it is known to form an alternative complex with PRC2, capable of weakly trimethylating H3K27. It has been demonstrated that PRC2-EZH1 and PRC2-EZH2 complexes are recruited for the same set of target genes. However, EZH1 appears to be more expressed in already differentiated adult cells while EZH2 expression is associated with proliferation [51]. During muscle differentiation, several studies have shown a role for EZH1 in transcriptional activation [60, 61]. In contrast, in ES cells it has been shown that EZH1 plays a redundant role in the execution of pluripotency during differentiation [53].

The genetic strategy of inhibition of EZH2 expression is useful to understand the function of the gene. A recent study [62] demonstrated that EZH2 inhibition is able to reverse the tumor phenotype of embryonic rhabdomyosarcoma RD cell lines and in alveolar rhabdomyosarcoma cell lines [63], even in the presence of proliferative stimuli, such as the addition of serum to growth conditions. Inhibition of EZH2 restores the myogenic phenotype through derepression of the myogenin and muscle creatine kinase (MCK) genes. Similar results were obtained by transfecting RD cells with an siRNA targeting the 5′UTR of the gene encoding for endogenous EZH2 [64] confirming the effects caused by EZH2 silencing.

Another approach used is to stably transfect RD cells with a lentiviral vector expressing a short hairpin RNA (shRNA) targeting EZH2. The lentiviral vector induced the silencing of EZH2 and consequently the depression of genes encoding for p21, myogenin, and muscle creatine kinase and the formation of MHC-positive polynuclear fibers. Chromatin immunoprecipitation analysis showed that recruitment of EZH2 to the regulatory regions of muscle-specific, early (myogenin) and late (MCK, MHC) genes decreases in cells in which EZH2 is silenced. This correlates with a decrease in H3K27me3 levels at the indicated regulatory loci. These results suggest that silencing EZH2, in actively proliferating embryonic RMS cells, proved to be a successful strategy to promote their exit from the cell cycle and restoration of muscle differentiation [62].

## Pharmacological inhibition of EZH2

Some pharmaceutical companies have developed several molecules that can effectively inhibit PRC2 activity (Table 1) [65].

The first inhibitor, which has been predominantly used in basic studies to investigate the role and function of EZH2 is 3-Deazaneplanocin A (DZNep) [66]. It is an inhibitor of S-adenosyl homocysteine hydrolase, an enzyme involved in the methyl cycle, catalyzing the hydrolysis of S-adenosyl-L-homocysteine (SAH). The mechanism of action of this molecule has not yet been fully understood; it appears that the absence or reduction of S-adenosyl homocysteine hydrolase activity, and the consequent accumulation of S-adenosyl-L-homocysteine, leads to the inhibition of SAM-dependent methyltransferases. Therefore, the effect of DZNep in inhibiting histone methylation is not selective for EZH2 [67]. DZNep treatment induces significant antitumor activity in various cancers, corresponding to PRC2 inhibition and reduction of H3K27me3 levels in the target genes of the complex [67]. DZNep induces apoptosis in breast, colorectal, prostate, hepatocellular carcinoma, and rhabdomyosarcoma cancer [68].

**Table 1:** PRC2 inhibitors

Inhibitors of EZH2 methyltransferase activity	SAH hydrolase inhibitor which globally inhibits histone methylation	3-deazaneplanocin A (DZNep) [53]
	SAM-competitive Inhibitors	GSK126, EPZ005687, EL1 [70], GSK343, GSK926, Tazemetostat, EPZ011989, CPI-1205, CPI-169, ZLD1039, PF-06821497; [65, 67, 69, 71–73]; UNC199, OR-S1/OR-S2, DS-3201b [74, 75]
Inhibitors that break PRC2’s structure	Disrupting the EZH2-EED interaction	SAH-EZH2, Astemizole, Wedelolactone, apomorphine hydrochloride, oxyphenbutazone, nifedipine, ergonovine maleate, AZD9291, MAK683 (EED226) [76, 77]
	Disrupting the EZH2-SUZ12 interaction	A769662 (an AMPK agonist) [76]
Suppressing EZH2 through triggering EZH2 degradation	GNA022, ANCR, FBW7, ZRAMB1 siRNA and other inhibitors [77]	

12-O-tetradecanylphorbol-13-acetate (TPA) is another modulator of EZH2 activity. In hepatocellular carcinoma, it promotes the reduction of EZH2, EED, and H3K27me3 expression levels. In embryonal hepatocarcinoma and rhabdomyosarcoma cells, TPA treatment induces cell cycle arrest at the G0/G1 stages by a mechanism mediated by the PKCα and ERK pathways [78].

GSK126, discovered in 2012, is 1000-fold more selective for EZH2 than 20 other methyltransferases and 150-fold more selective than EZH1. This molecule has been tested on large B-cell lymphoma cells and rhabdomyosarcoma cell lines and induces a dose-dependent decrease in H3K27me3. Furthermore, GSK126 inhibits proliferation in vitro, the most sensitive cells being those in which EZH2 is mutated [79].

Another strategy to inhibit EZH2 involves blocking interactions with the other subunits of PRC2: SUZ 12 and EED, which are required for the catalytic activity of the complex. Several research groups have tested the efficacy of some of these molecules on embryonic rhabdomyosarcoma cell lines. The aim of these studies is to be able to inhibit the enzymatic activity of PRC2, through the downregulation of EZH2, resulting in the expression of muscle-specific genes silenced by the complex and restoration of myoblastic differentiation. To translate the results obtained by gene inhibition of EZH2, in the study reviewed previously [62], into a future potential clinical approach for the treatment of aggressive embryonic rhabdomyosarcoma, the feasibility of pharmacological inhibition of EZH2 in RD cells was evaluated. These were treated with the first-generation inhibitor 3-Deazaneplanocin A (DZNep), an inhibitor of S-adenosyl-homocysteine hydrolase that induces EZH2 degradation [64]. Two inhibitors of EZH2 catalytic activity, MC1948 and its derivative MC1945, were also tested in parallel [80]. The data show a significant reduction in proliferation rate in RD cells treated for 72 h and 96 h with DZNep or MC1945 (1 μM), compared with untreated cells. A more significant inhibition of cell proliferation was achieved by treatment of RD cells at higher concentrations (5 μM) of each molecule, suggesting a dose-dependent inhibitory effect. These effects were accompanied by down-regulation of EZH2 protein levels in DZNep-treated cells, whereas they remained constant after treatment with the catalytic inhibitor MC1945. Both DZNep and MC1945 treatment resulted in decreased global H3K27me3 levels. Whereas levels of H3K9me3, another EZH2-independent repressive mark, remained unchanged after both treatments, demonstrating the specificity of the two molecules for the EZH2-containing complex. Similar results were obtained in preliminary experiments using MC 1948. Taken together, these results clearly suggest that pharmacological inhibition of EZH2 affects the proliferative potential of embryonic RMS cells by mimicking the cellular effect induced by siRNA-mediated gene-specific inhibition of EZH2.

In a more recent study, the expression levels of EZH2 and its catalytic activity were examined in TPA-treated RD cells and in cells treated with TPA in combination with GSK126, a highly selective inhibitor of EZH2 catalytic activity [81]. The results of the study showed a significant morphological change in TPA-treated cells, which appeared larger and more elongated, phenotypically similar to myocytes. The study also showed in RD cells treated with the two molecules (TPA GSK126) in combination, morphological changes more profound than the treatment with the single molecule. RD cells appeared more elongated and organized in a myotube-like manner. Numerous polynuclear cells were observed in TPA- and GSK126-treated cells and induced differentiation, whereas only a few polynuclear cells were present in single-molecule treated cells. These results demonstrate that TPA only partially reduces the activity of the PRC2 complex. In addition, TPA treatment in combination with selective inhibition of EZH2 by GSK126 resulted in a synergistic effect toward terminal differentiation of RD cells.

## Conclusion

Understanding the transition of physiological/pathological mechanisms in skeletal muscle cell differentiation is critical to improve the current therapeutic strategies. Clarifying the mechanisms that lead to deregulation of the muscle differentiation process and tumor formation is therefore essential to find new targets and new therapies that can increase the chances of survival and minimize side effects. Epigenetic alterations play an important role in tumor development, since they are reversible and can be counteracted with specific inhibitors or enzymes. This peculiarity makes epigenetic markers an attractive target for therapeutic intervention in cancer [82]. This approach appears to be of particular interest in pediatric embryonic RMS, in which pathogenetic mechanisms involve deregulation of genes encoding for factors that regulate cell fate [83]. Polycomb repressive complexes regulate the transcription of numerous genes involved in development and differentiation. The catalytic subunit of the PRC2 complex has been observed to be overexpressed in rhabdomyosarcoma cell lines even under differentiating conditions [62]. These studies provided insight into the key role of EZH2 in the inhibition of skeletal muscle differentiation. Several molecules capable of inducing inhibition of EZH2 catalytic activity (TPA, GSK126, DZNep, MC1945, MC1948) have been tested in recent years in embryonic rhabdomyosarcoma cells. It was observed that some of these, alone or in combination with other EZH2 inhibitors, induce an antiproliferative effect in RD cells and restoration of terminal differentiation, demonstrated by the presence of polynuclear myotubes and expression of late muscle-specific genes. However, the use of these inhibitors has not yet led to improved therapeutic treatment of RMS. Indeed, there are currently no drugs able to inhibit the catalytic activity of EZH2 in the clinical field for the treatment of Rhabdomyosarcoma. Currently, research has led to the knowledge of the function of EZH2 in tumor progression [58]. The main obstacles of clinical applications are mainly related to tumor variability (histological subtypes, presence of metastases) and the need for a more accurate understanding of the regulatory mechanisms of the PRC2 complex. As described in this review, several EZH2 inhibitor molecules have been studied in various tumor cell lines. These findings, make the drug a potential candidate in anti-cancer therapy, however the indirect mechanism may affect many processes and makes further studies necessary to address its target specificity [84]. Therefore, initiation of a clinical strategy by specific EZH2 inhibitors must go through a complete understanding of the molecular aspects that characterize tumor initiation.

## Data Availability

Not applicable.

## Abbreviations

aRMS: Alveolar rhabdomyosarcoma
bHLH: Basic helical domain
CDKs: Cyclin-dependent kinases
CTD: Carboxy-terminal domain
DZNep: 3-Deazaneplanocin A
ERK: Extracellular signal-regulated kinases
eRMS: Embryonal rhabdomyosarcoma
ESCs: Embryonic stem cells
EZH1: Enhancer of zeste homolog 1
EZH2: Enhancer of zeste homolog 2
FGF: Fibroblast growth factor
H3K27me3: Lysine 27 methylation of histone H3
H3K9me3: Lysine 9 methylation of histone H3
MCK: Muscle creatine kinase
MCL: Light chains
MHC: Myosin heavy
MRF4: Myogenic regulatory factor 4
MRFs: Myogenic regulatory factors
OS: Overall survival
PcG: Polycomb group proteins
PKCα: Protein kinase C-alfa
PRC1: Polycomb repressive complex 1
PRC2: Polycomb repressive complex 2
RD: Embryonal rhabdomyosarcoma cell line
RMS: Rhabdomyosarcoma
RNA Pol II: RNA polymerase II
SAH: S-adenosyl-L-homocysteine
SAM: S-Adenosyl methionine
shRNA: Short hairpin RNA
siRNA: Small interfering RNA
STS: Soft tissue sarcoma
TPA: 12-O-tetradecanylphorbol-13-acetate
